# How to unveil self-quenched fluorophores and subsequently map the subcellular distribution of exogenous peptides

**DOI:** 10.1038/srep20237

**Published:** 2016-02-03

**Authors:** Jean-Marie Swiecicki, Frédéric Thiebaut, Margherita Di Pisa, Simon Gourdin -Bertin, Julien Tailhades, Christelle Mansuy, Fabienne Burlina, Serge Chwetzoff, Germain Trugnan, Gérard Chassaing, Solange Lavielle

**Affiliations:** 1 Sorbonne Universités, UPMC Univ Paris 06, LBM, 4, Place Jussieu, 75005 Paris, France; 2 Ecole Normale Supérieure – PSL research University, Département de Chimie, 24 Rue Lhomond, 75005 Paris, France; 3 CNRS, UMR 7203, LBM, Paris, France; 4 Sorbonne Universités, UPMC Univ Paris 06, PHENIX, 4 Place Jussieu, 75005 Paris, France; 5 CNRS, UMR 8234, PHENIX, Paris, France; 6 INSERM-ERL 1157, CHU Saint Antoine, 27 rue de Chaligny, 75012 Paris, France; 7 AP-HP, Hôpital Saint Antoine, 75012 Paris, France; 8 INRA, UR892, Virologie et Immunologie Moléculaires, 78350 Jouy-en-Jossas, France

## Abstract

Confocal laser scanning microscopy (CLSM) is the most popular technique for mapping the subcellular distribution of a fluorescent molecule and is widely used to investigate the penetration properties of exogenous macromolecules, such as cell-penetrating peptides (CPPs), within cells. Despite the membrane-association propensity of all these CPPs, the signal of the fluorescently labeled CPPs did not colocalize with the plasma membrane. We studied the origin of this fluorescence extinction and the overall consequence on the interpretation of intracellular localizations from CLSM pictures. We demonstrated that this discrepancy originated from fluorescence self-quenching. The fluorescence was unveiled by a “dilution” protocol, *i.e*. by varying the ratio fluorescent/non-fluorescent CPP. This strategy allowed us to rank with confidence the subcellular distribution of several CPPs, contributing to the elucidation of the penetration mechanism. More generally, this study proposes a broadly applicable and reliable method to study the subcellular distribution of any fluorescently labeled molecules.

The subcellular distribution of bioactive molecules is routinely explored by fluorescence microscopy, including confocal laser scanning microscopy (CLSM)^1^,^2^. CLSM allows studying the subcellular localization of fluorescent molecules within living cells with excellent time (spinning-disk confocal microscopes) or spatial resolution (stimulated emission depletion microscopy, STED)^3^,^4^,^5^. CLSM is intrinsically based on the fluorescence properties of the molecules, requiring their derivatization with a dye or their fusion with a fluorescent protein. Besides the physicochemical perturbation induced by the addition of a fluorophore to the native molecule^6^, numerous technical pitfalls have been reported^7^.

Cationic cell-penetrating peptides (CPPs) are short arginine-rich sequences, which penetrate cells and facilitate the cellular uptake of cargoes covalently linked or non-covalently associated (the sequences of three cationic arginine-rich CPPs are reported Supplementary Table 1)^8^,^9^,^10^. There is now abundant and consistent evidences obtained by various experimental protocols that CPPs can penetrate *via* energy-dependent routes (endocytosis) as well as *via* energy-independent pathways (“direct-translocation”)^11^,^12^,^13^. The question of the penetration mechanisms is fundamental for the development of CPPs as delivery vectors, because the endosomal route might result in endosomal entrapment or lysosomal degradation of the payload. Unraveling the subcellular distributions of a CPP and of its cargo is crucial to understand the mechanism of internalization, and also to select the best system for delivery to the targeted subcellular compartment. Most of the properties of CPPs are described thanks to CLSM images, as they penetrate silently into living cells^14^,^15^,^16^,^17^. CLSM offers an easy readout to map their intracellular localization, which often, if not always, correlates with their internalization pathways. Fluorescence-based analytical tools applied to CPPs have been first criticized because of the redistribution of CPPs upon fixation of the samples^18^. As a consequence, CLSM has to be performed with living cells. However, this artifact is not the only one.

Alternatively, a quantitative mass-spectrometry procedure allows the precise determinations of the amount of internalized CPP and of CPP associated on the outer leaflet of the cell membrane. With this assay, a ~100 times larger quantity of CPP on the membrane than inside the cell was systematically measured (corresponding to ~1–10 μM intracellular concentration depending on the CPP)^11^,^12^. Repeated washing steps were not sufficient to remove membrane-bound CPPs, enzymatic digestion or chemical treatment to modify the extracellular peptide being required^19^. These data corroborate the high affinity of CPPs for anionic phospholipids and anionic polysaccharides of the extracellular matrix (Supplementary Table 1)^20^,^21^. In contrast, direct observation of living cells after incubation with 5–20 μM fluorescently labeled CPPs does not show any membrane-associated fluorescence^18^,^22^,^23^,^24^. We hypothesized that this lack of fluorescence may come from fluorescence self-quenching, as membranes were crowded by CPP leading to a high surface density of the fluorophore^25^,^26^,^27^,^28^,^29^. This observation might seem anecdotic but fluorescence self-quenching must also appear in other subcellular regions and consequently, bright cellular compartments in CLSM images might contain less fluorescently tagged peptides than some dark regions of the cell, leading to incorrect interpretation of the data.

Herein, we demonstrate that fluorescence self-quenching is a frequent phenomenon for the CPPs derivatized by the most widely used fluorophores (fluorescein or rhodamine). Fluorescence self-quenching occurs in regions where CPPs are densely packed and results in significantly lower than expected fluorescence intensities. We further develop a simple but particularly efficient method, which reduces the interactions between the fluorophores and reveals the presence of concentrated peptides. This procedure allows both characterizing the subcellular distribution of CPPs upon internalization and getting insights into the entry mechanisms.

## Results

### Fluorescence is self-quenched in the presence of analogues of cell membrane or cell matrix constituents

**Figure 1:**
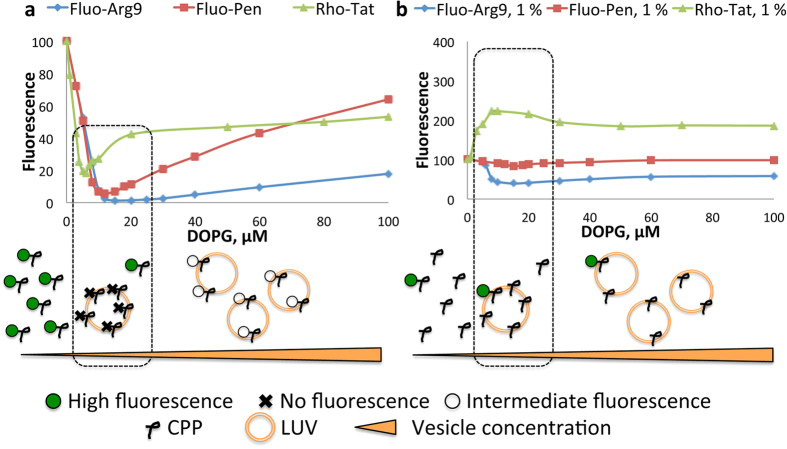
Fluorescence self-quenching in the presence of membranes. Titration of fluorescent CPPs by LUVs (100 nm) made of DOPG. The fluorescence intensity at the maximum is measured. The wavelengths of the maxima change only very weakly over the course of the titration (<2 nm). Below is depicted the evolution of the fluorescence of individual fluorophores bound to CPP in the presence of increasing concentrations of LUVs. (**a**) Titration of fluorescent CPP (500 nM). (**b**) Titration of 1% of fluorescent CPP (5 nM) in the presence of a large excess of its acetylated analogue (99%, 495 nM).

The fluorescence properties of Pen (Penetratin), Tat and Arg9 (nona-arginine cell-penetrating peptide) labeled with fluorescein or rhodamine B were first examined in the presence of large unilamellar vesicles (LUVs) made of the anionic phospholipid (PL) 1,2-di-oleoyl-*sn*-glycero-3-phospho-(1′-*rac*-glycerol) (DOPG) (In our study, peptides were derivatized at the *N*-ter with fluorescein on the side chain of an extra lysine or with a rhodamine B azide derivative clicked to the side-chain of an extra propargylglycine; the chemical structures are reported Supplementary Figs 1 and 2, and the syntheses are described in the Supplementary Note 1). The titration by LUVs of the fluorescent CPPs showed a dramatic dependence of the fluorescence on the concentration of PLs (Fig. 1a). Strong fluorescence drops (~20- to 100-fold) were observed for all fluorescent peptides with increasing concentrations of DOPG, with a minimum for a CPP/PL ratio ~1:20, corresponding to the neutralization of charges between cationic CPPs and anionic PLs from the outer leaflet of LUVs. For this ratio, fluorescent CPPs are quantitatively adsorbed and saturate the vesicle surface (Scheme in the Fig. 1a).

These fluorescence decreases were not solely related to the binding of CPP to the negatively charged bilayer (change of the surrounding medium), as the fluorescence was partially restored upon addition of a large excess of vesicles (which resulted in reduced concentration of the fluorescent peptide at the surface of LUVs). Alternatively, the “inner filter effect” is often cited to rationalize the fluorescence drop observed in the presence of an increasing concentration of fluorescent dye. The inner filter effect corresponds to the re-absorption of the fluorescence by a solution, which is too concentrated in a fluorophore whose excitation and emission spectra overlap. First, it is important to notice that the fluorescence emission intensity of rhodamine- or fluorescein-labeled CPPs in plain buffer is linear below ~1 μM. Since the inner-filter effect would induce a loss in fluorescence linearity, it is negligible at 500 nM. In the presence of phospholipids, the global concentration of the dye does not change and the absorbance of the sample remains similar. As a consequence, the inner-filter effect from the solution is also negligible. The local concentration of the fluorescent peptide on the membrane does also not induce a local inner-filter effect. Indeed, a photon emitted by a fluorescent peptide adsorbed on a vesicle encounter, in average, at least one vesicle (the justification is presented in the Supplementary Note 2). The first vesicle, from which the photon originates, does not absorb more fluorescence than the other vesicles in solution that the photon encounter. Because the solution does not generate a significant inner-filter effect, the first vesicles does not create a significant local inner-filter effect either. In conclusion, the hypothesis that the observed fluorescence quenching could originate from the inner-filter effect has to be ruled out. Fluorescence drops are therefore likely to find their origin in the interactions between the fluorophores, leading to fluorescence self-quenching.

Fluorescence self-quenching has already been described for biomacromolecules that are labeled with multiples copies of the same fluorophore: their brightness is not proportional to the extend of the labeling^30^. The self-quenching has been attributed to the clustering of the fluorophores that occurs during the labelling process and results in an aggregation of the fluorophores bound to the biomacromolecule^31^,^32^. Indeed, the formation of aggregates is an important cause of fluorescence quenching for most aromatic π-conjugated hydrocarbons (some non-planar hydrocarbon have an enhanced fluorescence emission upon aggregation)^33^.

The fluorescence self-quenching that we observed with fluorescently labeled CPPs cannot be rationalized by the usual dynamic or static quenching phenomenological phenomena, because the experimental curves did not follow the phenomenological model of Stern and Volmer^34^ (Supplementary Note 2 and Supplementary Fig. 3). One possible explanation is thus the formation of “dark dimers” (or “dark aggregates”) of fluorophores, that corresponds to the model of static quenching, combined with dynamic quenching between single fluorophores and the dimers (or aggregates). This mechanism of fluorescence self-quenching has already been proposed to rationalize the self-quenching of carboxyfluorescein (and is discussed in the Supplementary Note 2)^35^.

Similarly, a high fluorescence self-quenching was also observed during the titration of fluorescently labeled CPPs with dextran sulfate, a polysulfated carbohydrate that mimics the glycosaminoglycan rich environment of the cell matrix (Supplementary Fig. 4), as observed by Ziegler and Seelig with heparin^36^. Dextran sulfate also concentrated the CPPs and induced a spatial proximity between the fluorophores. Using both dextran sulfate or DOPG as CPPs interaction partners, we noticed that the self-quenching behaviors of peptides labelled either with fluorescein or with rhodamine B were qualitatively very similar (Fig. 1a and Supplementary Fig. 4). This suggests that the mechanisms underlying self-quenching of CPPs are similar for both fluorophores.

### “Diluting” fluorescent peptides prevents fluorescence self-quenching

The hypothesis that the fluorophores were the quenchers was further confirmed by “diluting” the fluorescent peptide with its acetylated analogue. Fluorescence quenching was indeed dramatically reduced by this dilution procedure, the fluorescence varying by only a factor ~0.5 to 2.5 over the titration (Fig. 1b and Supplementary Fig. 4). This demonstrate that the change of polarity or viscosity of the surrounding medium upon binding does not induce the observed fluorescence self-quenching. These small residual variations might be attributed to subtle differences in the environment of the fluorophore. All these data reinforced the hypothesis that fluorescence self-quenching might be a general phenomenon arising in each situation where fluorescent molecules are densely packed, such as CPP on cell surface or in subcellular regions where they are concentrated.

### The “dilution” method reveals fluorescence self-quenching on cell surface

**Figure 2:**
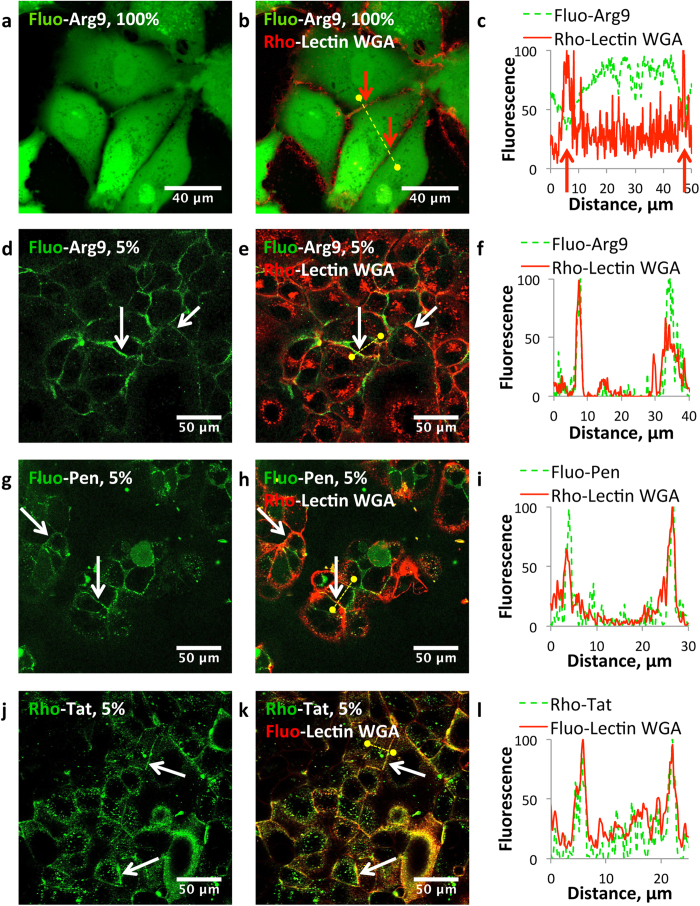
“Dilution” of the fluorescent CPP by its acetylated analogue reveals plasma membrane bound CPP. Unfixed MA-104 or Caco-2 cells were visualized by CLSM after 10 min incubation with 20 μM CPP (in green) and a fluorescent Lectin WGA (0.02 mg.mL^−1^, red) in Opti-MEM. Cells were washed with Opti-MEM prior to observation. (**a**–**c**) MA-104 cells incubated with 20 μM Fluo-Arg9 and Rho-Lectin WGA. (**d**–**f**) MA-104 cells incubated with Fluo-Arg9 (5%, 1 μM) in the presence of a large excess of its acetylated analogue (95%, 19 μM) and Rho-Lectin WGA. (**g**–**i**) Caco-2 cells incubated with Fluo-Pen (5%, 1 μM) in the presence of a large excess of its acetylated analogue (95%, 19 μM) and Rho-Lectin WGA. (**j**–**l**) Caco-2 cells incubated with Rho-Tat (5%, 1 μM) in the presence of a large excess of its acetylated analogue (95%, 19 μM) and Fluo-Lectin WGA. Pictures (**a**,**d**,**g**,**j**) feature only the fluorescence of the peptide, whereas pictures (**b**,**e**,**h**,**k**) are overlays of the fluorescence of the CPP and of the Lectin WGA. (**c**,**f**,**i**,**l**) show the intensity profiles of the fluorescence from the CPP and from the Lectin WGA along the yellow dotted line on the picture in the same row.

**Figure 3:**
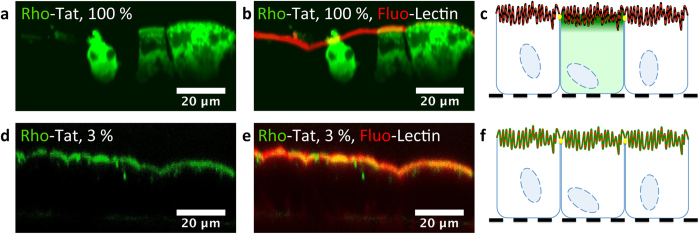
“Dilution” of Rho-Tat by its acetylated analogue reveals the CPP located on the brush border of differentiated Caco-2 cells. Unfixed Caco-2 cells grown over 21 days on Transwell permeable supports were incubated with 20 μM Tat (in green). Tat was placed only in the compartment above the cells over 10 min in the presence or not of its acetylated analogue. The fluorescein-labeled Lectin WGA was added both in the compartment above and below the cells (in red). Cells were washed with Opti-MEM prior to observation. (**a**–**c**) Cells were incubated with Rho-Tat (20 μM). (**d**–**f**) Cells were incubated with Rho-Tat (3%, 600 nM) in the presence of large excess of its acetylated analogue (97%, 19.4 μM). Pictures a and d feature only the fluorescence of the peptide whereas picture b and e are overlays of the fluorescence of the CPP and of the Lectin WGA. Scheme c and f are interpretations of the pictures b and e respectively. At a 20-μM concentration of Rho-Tat, the membrane-associated fluorescence of Tat is quenched (in black) whereas at high dilution, the presence of Tat on the microvilli is revealed (in green). At such high dilution, the intracellular presence of Tat is not detectable. The presence of the tight-junction (in yellow) explains the regionalization of the fluorescence when the peptide is only incubated on the top of the cells. The filter is figured as a black dotted line.

This “dilution” protocol was subsequently applied to living cells to shed light on the presence of eventual self-quenched regions (MA-104 cells, epithelial monkey kidney cells and Caco-2 cells, epithelial cells from a human colorectal adenocarcinoma; viability assay, see Supplementary Note 3 and Supplementary Fig. 5). Cells were incubated with a CPP concentration (20 μM) fitting into the common concentration range used in the field of CPPs^22^,^37^,^38^. We anticipated that the use of different (fluorescent CPP)/(total CPP) ratios would be necessary depending on the local concentration of the CPP, densely crowded regions requiring higher dilution than less crowded compartments. We first incubated cells with 100% fluorescently labeled CPP (Fig. 2a–c and Supplementary Fig. 6). Similarly to the pictures usually presented in the literature, we did not observe any membrane-associated fluorescence (the membrane are fluorescently stained using fluorescein or rhodamine-labeled Lectin WGA, a protein that binds cell surface glycans)^18^,^22^,^23^,^24^. We then performed high-dilution experiments (1–5% fluorescent CPP, with the total concentration of CPP always fixed at 20 μM, *i.e*. dilution with 95–99% acetylated CPP analogue) to reveal the regions having the highest CPP local concentrations (Fig. 2d–l). The presence of the fluorescent CPPs on the plasma membrane became visible and colocalized with Lectin WGA. The brightest regions of the cells were systematically the plasma membrane contrasting strongly with the observation made with 100% fluorescent CPP. At a dilution of ~1% (200 nM of fluorescent CPP), the fluorescence was just above the detection limit, whereas at ~20%, fluorescence was no longer detectable on the plasma membrane because of self-quenching. Importantly, the phenomenon of fluorescence quenching was also observed when cells where incubated with a low concentration (1 μM) of 100% fluorescent CPP. Similarly, the fluorescence of the CPPs associated to membranes could be recovered by the addition of the acetylated analogue (19 μM) to the incubation medium containing 1 μM of the fluorescently labeled peptide (Supplementary Video 1). These observations were further extended to Caco-2 differentiated cells that morphologically and functionally resemble the enterocytes of the small intestine (Fig. 3)^39^. CPPs were incubated over 10 min on the apical pole of these polarized cells. With 20 μM of fluorescent CPP, only the cytosol is fluorescent whereas at 3% dilution, the apical pole is highly fluorescent and colocalize with the fluorescein-labeled Lectin WGA. This shows that the fluorescence of the CPP located on the surface of the brush border is quenched when high concentration of fluorescent CPP is used. All these data demonstrated that the plasma membrane corresponded to the highest local density of CPPs, in agreement with the quantitative mass spectrometry measurements.

### Peptide-rich lysosomes can be non-fluorescent

**Figure 4:**
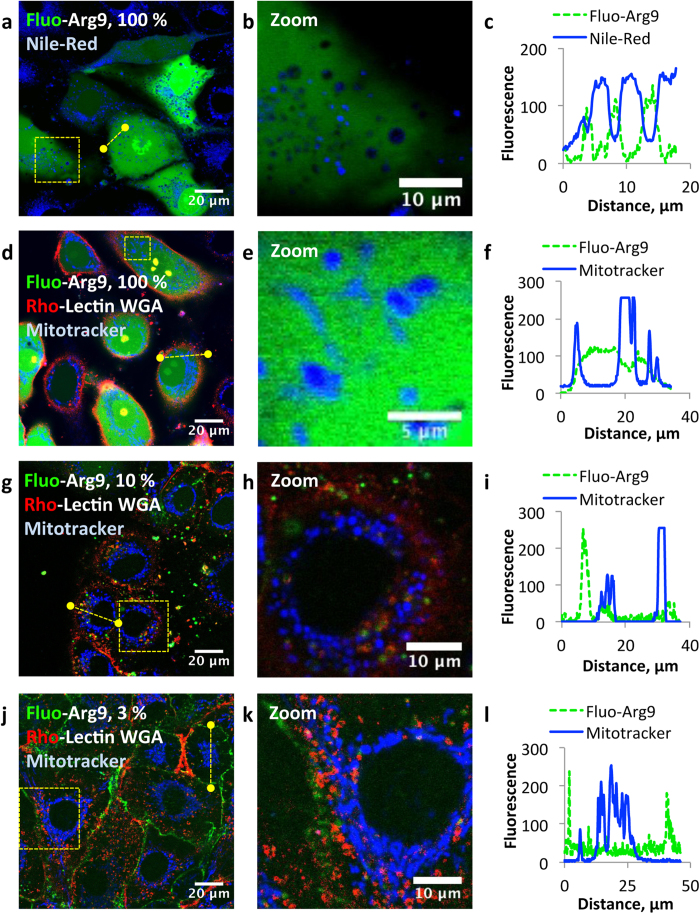
Lipid droplets and mitochondria colocalize with dark dots into the cytoplasm and correspond to Arg9-poor regions. Unfixed MA-104 cells were visualized by CLSM after 10 min incubation with Fluo-Arg9 (in green) in Opti-MEM in the presence of specific staining fluorescent dyes. (**a**–**c**) Cells were incubated with 20 μM Fluo-Arg9. Lipid droplets were stained by Nile-Red (in blue). Due to spectral overlap with the CPP or with Nile-Red, cells were not co-incubated with a fluorescent Lectin WGA. (**d**–**l**) Cells were visualized by CLSM after 10 min incubation with 20 μM Arg9 in Opti-MEM in the presence of Rho-Lectin WGA (in red) and mitotracker deep-red (in blue). Arg9 was either 100% (**d**–**f**), 10% (**g**–**i**) or 3% (**j**–**l**) labeled with fluorescein. (**a**,**d**,**g**,**j**) are overlays between the fluorescence of the peptide, of the Lectin WGA (if applicable) and of the organelle staining agents. (**b**,**e**,**h**,**k**) are enlarged views of the yellow square drawn on the picture (**a**,**d**,**g**,**j**), respectively. (**c**,**f**,**i**,**l**) are intensity profiles of the green and blue fluorescence along the yellow dotted line of the picture (**a**,**d**,**g**,**j**), respectively.

We then analyzed some key organelles, which might correspond to the black dots observed while incubating cells with 100% fluorescent CPPs, *i.e*. lipid droplets, mitochondria and lysosomes (Fig. 2a). Under such conditions, we found that lipid droplets and mitochondria compartments colocalized with black dots after 10 min incubation (colocalization with specific staining fluorescent dyes, Nile-Red for lipid droplets, Mitotracker deep-red (Invitrogen) for mitochondria, Fig. 4). Whereas the hydrophobic lipid droplets were expected not to contain any polycationic CPP, it was not clear whether mitochondria might be concentrated in CPP or not. Indeed, some penetrating peptides were shown to cross both the plasma membrane and the mitochondrial inner membrane^40^. MA-104 cells were incubated with diluted fluorescent CPP (3 to 10%, Arg9, Fig. 4). Whatever the conditions, the mitotracker never colocalized with the fluorescence of CPP, showing that these compartments were not targeted by Arg9, in agreement with previously published data^41^.

**Figure 5:**
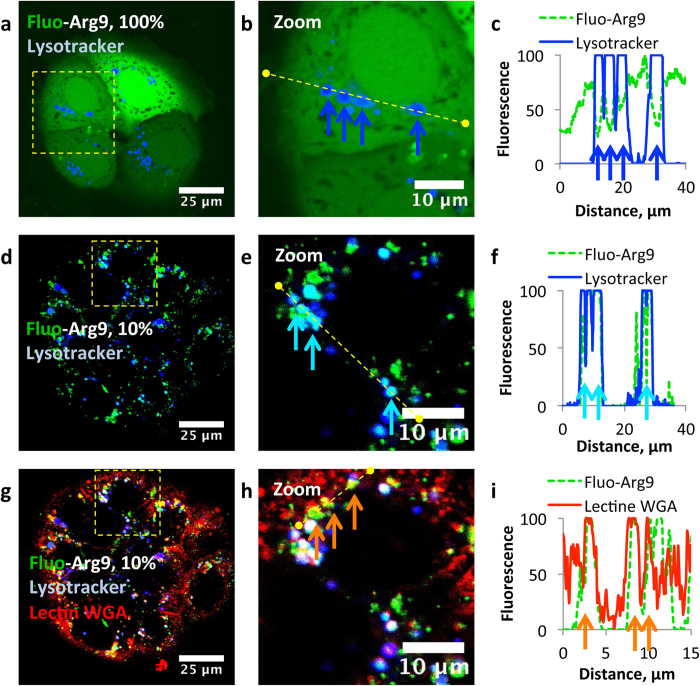
The dilution protocol at work: the example of Arg9. Unfixed and undifferentiated Caco-2 cells were visualized by CLSM after 60 min incubation in the presence of 100% (**a**–**c**) or 10% Fluo-Arg9 (**d**–**i**) (*i.e*. 20 μM or 2 μM Fluo-Arg9 plus 18 μM Arg9, in green) in Opti-MEM, of Rho-Lectin WGA (in red) and of lysotracker deep-red (in blue). Cells were washed with Opti-MEM prior to observation. (**b**,**e**,**h**) are respective enlarged views of the pictures (**a**,**d**,**g**) (regions framed by a yellow square). (**c**,**f**,**i**) are fluorescence intensity profiles for the CPP, the lysotracker or the Lectin WGA along the yellow cross-section of the picture in the same row. The blue arrows point towards subcellular regions, which are only labeled by lysotracker. The cyan arrows point toward subcellular regions, which exhibit both blue and green fluorescences. The orange arrows point toward subcellular regions, which exhibit both red and green fluorescences.

Lysosomes being associated with the endosomal pathway, it is fundamental to determine whether they are CPP-rich or not to better characterize the CPP internalization mechanism. Because the acidification process of endosomes to lysosomes is a slow process (~30 min), it was plausible that after 10 min incubation, lysosomes remained empty. Consequently, cells were incubated for 60 min with 100% Fluo-Arg9. The same subcellular distribution of the fluorescence was observed, and this fluorescence never colocalized with the fluorescence of the lysotracker (deep-red lysosomal fluorescent stain) (Fig. 5a–c; blue dots in the cell cytoplasm). In contrast, by using a 10% dilution, a strong colocalization between the lysotracker and the fluorescence of the CPP was observed (Fig. 5d–f; cyan dots inside the cytosol). This demonstrates that lysosomes may be sufficiently Arg9-rich organelles for the fluorescence to be self-quenched. Simultaneously, this also demonstrates that the complete fluorescence quenching of Fluo-Arg9 (Fig. 5a–c) does not originate from the low pH inside these lysosomes. At this dilution, all the fluorescence coming from Fluo-Arg9 was not systematically colocalized with the lysotracker dye (Fig. 5e). This non-lysosomal fluorescence frequently appears as yellow dots (Fig. 5g–i), corresponding to a colocalization with Lectin WGA, thus suggesting that Fluo-Arg9 followed an endocytic route, as previously shown for Lectin WGA^42^. We repeated this 60 min incubation time with Fluo-Pen, Fluo-Tat and Rho-Tat. When these fluorescently labeled peptides where incubated at high concentration, a diffuse cytosolic fluorescence as well as a punctuated fluorescence were observable (Supplementary Fig. 7). Interestingly, a partial colocalization between the bright fluorescent dots and the lysotracker dye was noticeable indicating that fluorescence was not totally self-quenched inside lysosomes. This is in agreement with lysosomes being less loaded by these CPPs than they were by Arg9, as previously demonstrated in other cell lines by quantitative mass spectrometry^12^. For Pen and Tat, endocytosis might thus be reduced, but remains an important internalization route as evidenced by the dotted fluorescence pattern observed after incubation in diluted conditions (10% fluorescent peptide, Supplementary Fig. 8). The reduced fluorescence self-quenching for Pen and Tat might also find its origin in differences between the fluorescence properties of fluorophores bound to various CPPs. In particular, the self-quenching propensities of fluorescein covalently associated to Tat or Pen is reduced in comparison to the one of fluorescein associated to Arg9, whose fluorescence deviates strongly from the Stern-Volmer self-quenching model in the presence of membranes (Supplementary Note 2 and Supplementary Fig. 3). Whatever the precise origin of these differences, the dilution protocol most fundamentally shows that the uptake of these three cationic CPPs proceeds essentially *via* an energy-dependent pathway.

## Discussion

**Figure 6:**
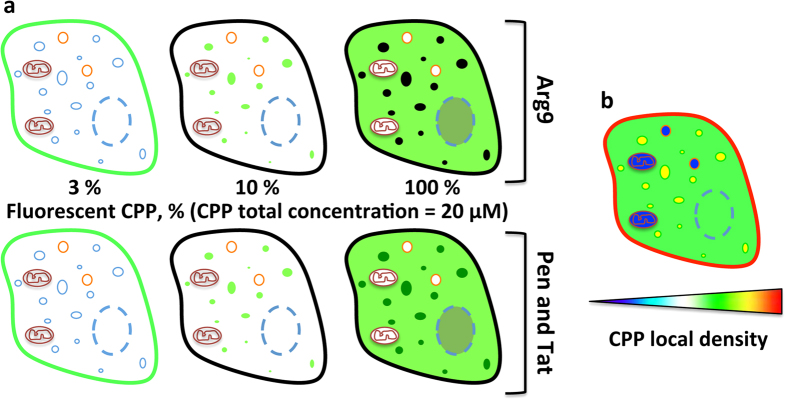
Mapping the subcellular distribution of CPPs. (**a**) Subcellular distribution of the fluorescence of the CPP labeled with fluorescein (total concentration 20 μM, in green) depending on the dilution. At high dilution (3%), only the plasma membrane is fluorescently stained by the CPP. At intermediate dilution (10%), a punctuated fluorescence is systematically observed, whatever the considered CPP. With 100% fluorescent CPP, the cell cytosol exhibits a high fluorescence. But regarding the lysosomes, two patterns can be distinguished: they are either highly fluorescent (Pen and Tat) or the fluorescence of the concentrated CPP is self-quenched (Arg9). (**b**) Recapitulation of the subcellular distribution of CPP as deduced from the dilution experiments: CPP are mostly adsorbed on the plasma membrane (red), locate in lysosomes (yellow) and are also present in the cytosol (green). Lipid droplets and mitochondria do not contain Arg9 CPP (blue).

Our method addresses a key issue in fluorescence imaging: the relationship between the local intensity of the fluorescence signal and the local concentration of the fluorescent probe. In particular, we demonstrated that the absence of fluorescence might be due to the self-quenching of the probe. To tackle recurring intramolecular self-quenching issues, chemical modifications of fluorescent scaffolds have been reported^32^,^43^. These methods have the drawback to require the tedious synthesis of new dyes. To tackle intermolecular self-quenching, we proposed herein a general and reliable method: to substitute in part the fluorescently labeled peptide by its non-fluorescent analogue. Depending on the ratio fluorescent/non-fluorescent peptides, the presence of the peptide is unveiled in different subcellular regions of the cell (with the total concentration being constant, 20 μM, Fig. 6a). Therefore, the distribution of an exogenous peptide should never be solely deduced from CLSM pictures obtained with 100% fluorescently labeled peptide. Under such experimental conditions, only the weakly to moderately concentrated subcellular regions are visible. It is noteworthy that such pitfall can explain several apparent contradictions in the field of cell-penetrating peptides regarding their internalization mechanisms.

Thanks to the dilution strategy of the fluorescent CPP by its acetylated analogue, it was not only possible to determine if the CPP is present in a cell compartment but also to rank the different regions of the cell depending on their relative concentration in CPP. As observed by the quantitative mass spectrometry assay, cationic CPPs rapidly crowd the plasma membrane surface. We unambiguously demonstrated that this molecular crowding is such that it induces the self-quenching of the fluorescent labels. We further investigated the intracellular distribution of CPPs: at 20 μM, they are concentrated into lysosomes and are, to some extent, localized into the cytosol and the nucleus, but neither in mitochondria nor in lipid droplets (Fig. 6a). Very interestingly, the different fluorescence patterns observed after incubation with 100% labeled CPPs, Arg9, Tat and Pen (Fig. 6a), may actually result from a similar relative distribution (Fig. 6b). In conclusion, at this concentration, endocytosis is the major internalization pathway for these three cationic CPPs, while a non-negligible fraction of the CPP is located in the cytosol.

More generally, fluorescence self-quenching may arise with any fluorescently labeled small molecules as soon as they are densely packed in subcellular regions of the cell. Such phenomenon might lead to erroneous interpretations of CLSM data. We propose an easy strategy to correlate the observed fluorescence to the amount of the molecules, unveiling with confidence the localization of the studied small-molecule.

## Methods

### Evaluation of fluorescence self-quenching in the presence of LUVs or of dextran sulfate

#### Origin and nature of the lipids, dextran sulfate and buffer used in this study

DOPG was purchased from Avanti Polar Lipids and delivered as sodium salt solutions in chloroform. Dextran sulfate (as a powder, from *Leuconostoc mesenteroides*, >500 kDa) was purchased from Sigma-Aldrich. Milli-Q water was produced using a Milli-Q Integral water purification system (Merck Millipore; the electrical resistivity of the water was measured and was always above 18 MΩ.cm). Phosphate buffered saline (PBS) buffer was supplied by Sigma-Aldrich as tablets yielding 10 mM phosphate buffer containing 2.7 mM potassium chloride and 137 mM sodium chloride.

#### Preparation of symmetric LUVs

DOPG as sodium salt in chloroform (25 mg.mL^−1^, 319 μL) was placed in a round-bottomed flask and 10 mL chloroform was added to increase the total volume (the lipid film formed upon evaporation is more spread on the surface of the round-bottomed flask if the initial volume is large). Chloroform was slowly evaporated under *vacuum* at 40 °C using a rotary evaporator and then placed 4 h under high-*vacuum*. The lipids were hydrated using PBS buffer. The turbid suspension of multilamellar vesicles was subsequently extruded 7 times through a 200 nm polycarbonate track-etch membrane and 10 times through a 100 nm polycarbonate track-etch membrane (Whatman) using a 10 mL Thermobarrel extruder (Lipex Biomembranes). The LUV solution was used from the day after their preparation and within one week. They were stored at 4 °C.

#### Preparation of stock-solutions of dextran sulfate

The stock solution of dextran sulfate was prepared in order to have an equivalent concentration of 500 μM in sulfated monomer; this has been evaluated by considering that each monomer is sulfated twice, thus having a molecular weight of 366 g.mol^−1^. As a consequence, the stock solution of dextran sulfate has a mass concentration of 183 mg.L^−1^.

#### Preparation of stock-solutions of peptides

For peptides containing a tryptophan residue, a rhodamine or fluorescein fluorophore the concentration was determined by UV-visible spectrophotometry. Spectra were recorded from solution of peptides in PBS buffer in quartz cuvettes with a Cary 300 Bio UV-Visible spectrophotometer (Varian). The concentrations were deduced using the Beer-Lambert’s law. For peptide, which does not contain any chromophore, the peptide was weighed out. Peptide quantities were deduced from the mean molecular weight, taking into account the presence of the trifluoroacetate ions. Even if this procedure seems approximate, it gave reliable results if the peptide was weighed out immediately after lyophilization.

For all the experiments where the fluorescent CPP is partially substituted by its acetylated analogue, stock solutions were systematically prepared in water: x % fluorescently labeled peptide was mixed with 100-x % of its unlabeled counterpart (1% < x < 10%).

#### Fluorescence measurements

Fluorescence emission spectra and time-course fluorescence measurements were recorded on a Jasco Fluorescence Spectrophotometer at 25 °C (controlled by a Jasco MCB-100 mini circulation bath). As an excitation source a xenon lamp was used and the excitation and emission slid widths were set at 5 nm. To avoid peptide adsorption and subsequent fluorescence quenching, polymethacrylate cuvettes (Sigma) were used. During the whole measurement the solution was stirred at 800 rpm.

#### Titration of the peptides by anionic LUVs or by the anionic polymer dextran sulfate

The best reproducibility (standard error below 5%) was obtained by preparing a fresh mixture of dextran sulfate and fluorescent peptide in PBS for each fluorescence measurement, instead of performing a classical titration (slow equilibration).

The desired volume of LUVs or dextran sulfate was added in a 4-mL fluorimeter cuvette in PBS, yielding to final concentrations comprised between 5 nM and 100 μM. The fluorescently labeled peptide was added at 25 °C under continuous stirring (concentration arbitrarily set at 500 nM). The fluorescence was recorded at the appropriate excitation/emission wavelengths until a stable signal is observed (about 30s). The excitation/emission wavelengths, which have been used for each fluorophore, correspond to the maximum in emission intensity in PBS buffer (we did not observe any significant fluorescence shift induced by the presence of dextran sulfate or LUVs and the fluorescence was recorded using the same excitation/emission wavelengths than the ones used for the peptide in PBS). Blank measurements were performed with various quantities of dextran sulfate or LUVs and were systematically subtracted from the fluorescence signal. These background intensities are only not negligible in the high fluorescence self-quenching regime.

The mixture between labeled and unlabeled peptide was titrated under the exact same conditions than the one used for the titration of the 100% labeled peptide.

### Confocal laser scanning microscopy with fluorescent CPPs and living cells

#### Origin and nature of the culture dishes, reagents and material used in this study

Cell culture reagents were purchased from Life Technologies (Gibco). μ-slides 8-well standard bottom, ibiTreat, tissue culture treated (ibidi, 1 cm^2^ per well) were purchased from Biovalley and tissue culture-treated polycarbonate Transwell filters with a 24-mm diameter and a 0.4-mm pore size from Corning Costar France. Lectin WGA labeled with fluorescein or rhodamine B, Lysotracker deep red, Mitotracker deep red and Nile-Red were purchased from Life Technologies. Confocal microscopy acquisition was performed using an inverted SP2 setup (DM IRBE2 microscope equipped with a TCS SP2 detector; Leica). Live imaging was performed using a thermo-controlled inverted setup (DMI4000 B microscope equipped with a EMCCD quantEM 512SC detector; Leica) equipped with a spinning disk CSU22 (Yokogawa).

#### Cell culture

MA-104 and Caco-2 cells were maintained in Eagle’s minimal essential medium (EMEM), supplemented with 5 to 10% fetal calf serum and antibiotics in culture flasks at 37 °C under an atmosphere of 5% CO_2_ in air and 100% relative humidity.

MA-104 or Caco-2 cells were transferred the day before the experiments to 8-well μ-slides at the desired density (5.10^3^ cells.cm^−2^) and grown 24 h in DMEM at 37 °C under an atmosphere of 5% CO_2_ in air and 100% relative humidity.

Caco-2 cells were also used 15 days after reaching confluence (21 days of culture), and the medium was changed daily. In this case, to allow separate access to the media on the two sides of Caco-2 cell monolayers, cells were grown on Transwell filters in order to obtain a dense uniform monolayer morphologically similar to the enterocytes lining the small intestine.

#### Confocal laser scanning microscopy

Cells were washed twice with Opti-MEM and incubated 10 or 60 min with CPPs (20 μM) at 37 °C in 200 μL Opti-MEM. Pre- or co-incubation with various staining agents were performed as described in Table 1. Many different combinations were tested, provided that no spectral overlap occurred between the fluorophores.

The supernatant was finally discarded and the cells were washed three times with Opti-MEM. 200 μL of Opti-MEM were added to hydrate the cells during the experiment. Cells were immediately observed by confocal microscopy at room temperature. For the movie, cells were maintained in a controlled chamber (37 °C under an atmosphere of 5% CO_2_ in air). For the visualization of the fluorescently labeled CPPs, the excitation/emission wavelengths were set as followed: for fluorescein, λ_ex_ = 488 nm, λ_em_ = 492–538 nm; for rhodamine, λ_ex_ = 543 nm, λ_em_ = 552–618 nm.

## Additional Information

**How to cite this article**: Swiecicki, J.-M. *et al*. How to unveil self-quenched fluorophores and subsequently map the subcellular distribution of exogenous peptides. *Sci. Rep*. **6**, 20237; doi: 10.1038/srep20237 (2016).

## Supplementary Material

Supplementary Information

Supplementary Information

## Figures and Tables

**Table 1:** Incubation conditions with various staining agents that have been used to identify the plasma membrane, endosomes, mitochondria, lipid droplets and lysosomes.

**Cell line**	**Staining agent**	**Incubation**	**Excitation wavelengths (**λ, **nm)**	**Emission Wavelengths (**λ, **nm)**
MA-104	Fluorescein-Lectin WGA	0.02 mg.mL^−1^, 10 min	488	499–535
	Rhodamine-Lectin WGA	0.02 mg.mL^−1^, 10 min	543	557–627
	Nile-Red	600 nM, 15 min	543	590–697
	Mitotracker deep red	200 nM, 25 min	633	683–817
	LysoTracker deep red	100 nM, 30 min	633	675–817
Caco-2	Fluorescein-Lectin WGA	0.02 mg.mL^−1^, 30 min	488	499–535
	Rhodamine-Lectin WGA	0.02 mg.mL^−1^, 30 min	543	557–627
	LysoTracker deep red	100 nM, 30 min	633	675–817
