# Supplementary material for: How to unveil self-quenched fluorophores and subsequently map the subcellular distribution of exogenous peptides
Source: Sci Rep. 2016 Feb 3;6:20237. doi: 10.1038/srep20237 (PMC4738315; doi:10.1038/srep20237)
Supplement: Supplementary Information [file srep20237-s1.pdf]

Supplementary Information

How to unveil self-quenched fluorophores and subsequently map the subcellular distribution of exogenous peptides

Jean-Marie Swiecicki[1,2,3,*], Frédéric Thiebaut[1,2,3], Margherita Di Pisa[1,2,3], Simon Gourdin[4,5], Julien Tailhades[1,2,3], Christelle Mansuy[1,2,3], Fabienne Burlina[1,2,3], Serge Chwetzoff[1,6,7,8], Germain Trugnan[1,7,8], Gérard Chassaing[1,2,3], and Solange Lavielle[1,2,3]

[1]Sorbonne Universités, UPMC Univ Paris 06, LBM, 4, Place Jussieu, 75005 Paris, France; [2]Ecole Normale Supérieure – PSL research University, Département de Chimie, 24 Rue Lhomond, 75005 Paris, France; [3]CNRS, UMR 7203, LBM, Paris, France; [4]Sorbonne Universités, UPMC Univ Paris 06, PHENIX, 4 Place Jussieu, 75005 Paris, France; [5]CNRS, UMR 8234, PHENIX, Paris, France; [6]INSERM-ERL 1157, CHU Saint Antoine, 27 rue de Chaligny, 75012 Paris, France; [7]AP-HP, Hôpital Saint Antoine, 75012 Paris, France; [8]INRA, UR892, Virologie et Immunologie Moléculaires, 78350 Jouy-en-Jossas, France.

*To whom correspondence should be addressed. E-mail: jmsd@mit.edu

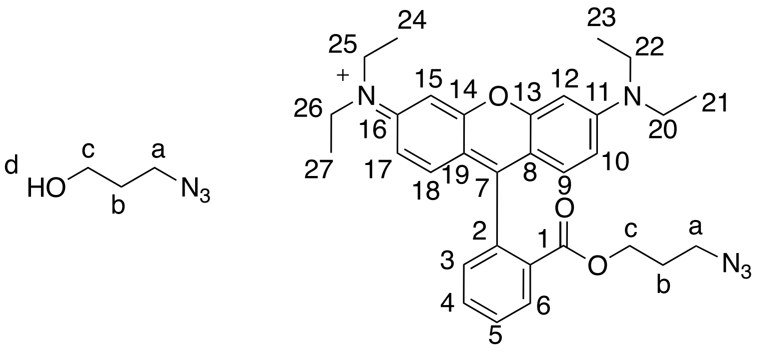

Supplementary Figure 1

Chemical structures of the azido linker and of the rhodamine B azide suitable for click reaction.

Structures of 3-azidopropan-1-ol (left) and of rhodamine B azide (right).

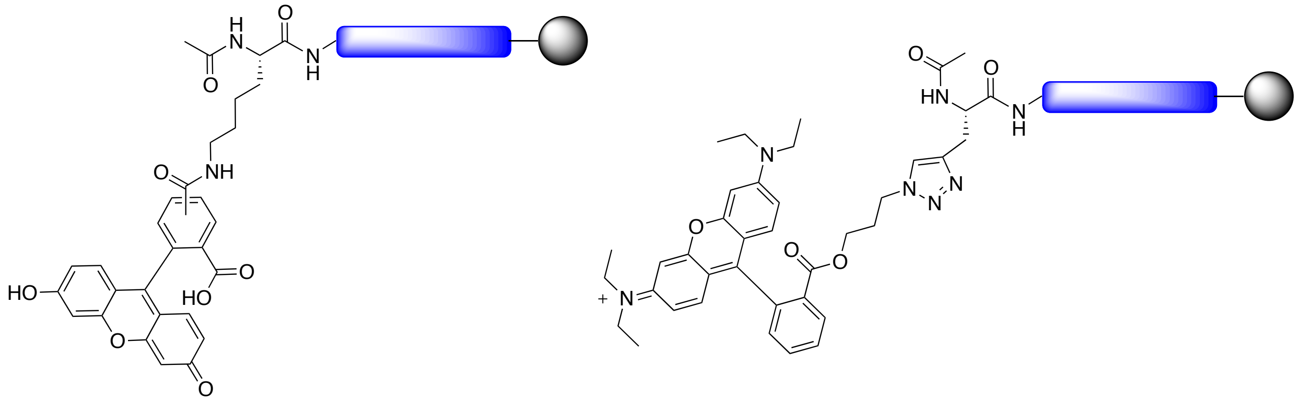

Supplementary Figure 2

General formula of the synthesized peptides bound to resin.

Structure of fluorescein (left) or rhodamine B azide (right) peptidyl-resins.

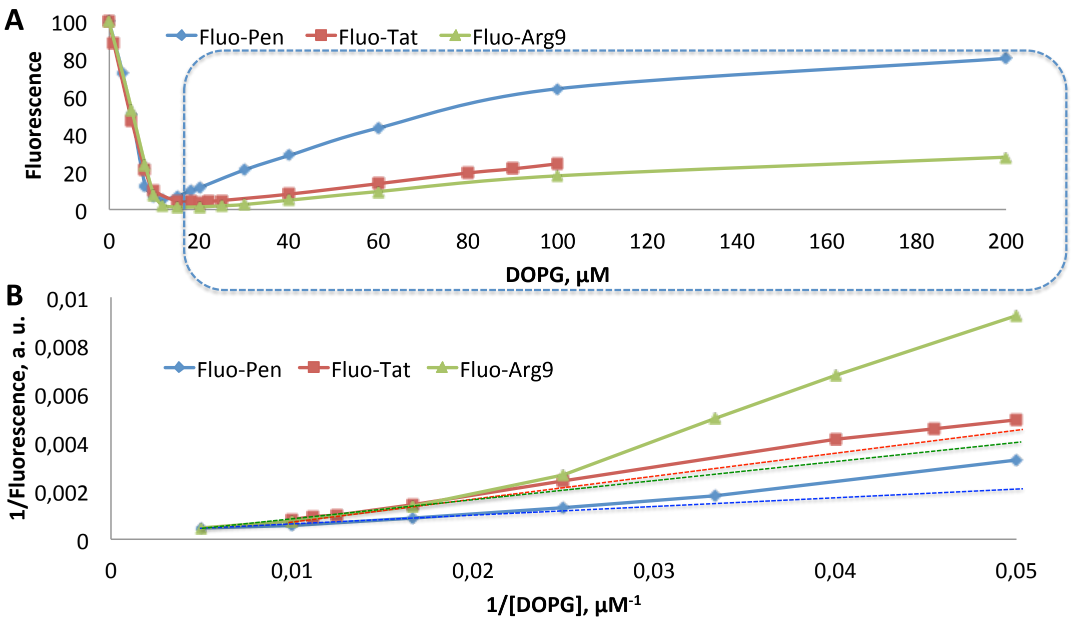

Supplementary Figure 3

The self-quenching of the fluorescently labeled peptide in the presence of vesicles does not follow the Stern-Volmer phenomenological model.

(A) Titration curves of the fluorescent CPPs by DOPG. (B) Stern-Volmer-type plots corresponding to the circled regions of graphic A. Dotted lines are linear fits of the three first points of each curve. They are "guides for the eyes" to see the upward curvatures of the Stern-Volmer plots.

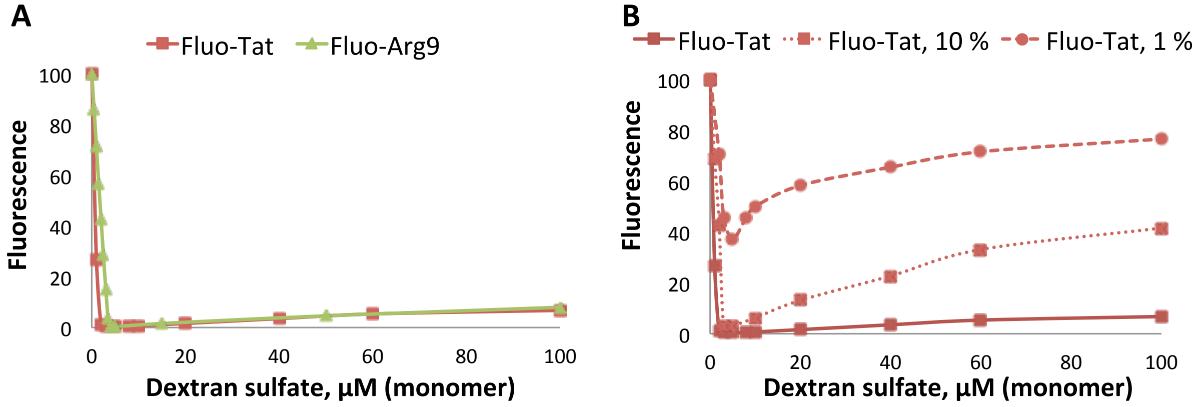

Supplementary Figure 4

Self-quenching and dequenching of fluorescent CPP in the presence of the anionic polysaccharide dextran sulfate.

(A) Titration of 500 nM of Fluo-Tat or Arg9 by dextran sulfate (5 nM-100 µM). (B) Titration of 1 or 10 % of Fluo-Tat (5 or 50 nM, respectively) in the presence of a large excess of its acetylated analogue (99 %, 495 nM or 90 %, 400 nM, respectively). The dilution protocol reduces gradually self-quenching. The variations of fluorescence due to the lineal density of peptide along the polymer are dramatically reduced.

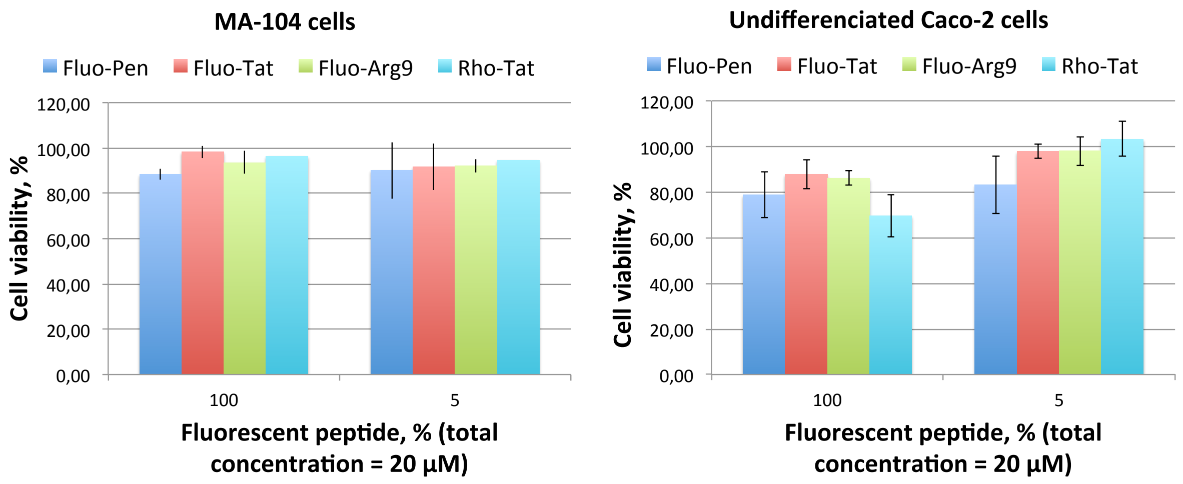

Supplementary Figure 5

Cell viability in the presence of fluorescent CPPs.

Viability of MA-104 cells (left) or Caco-2 cells (right) measured after 2 h incubation at 37 °C in the presence of fluorescent peptides. The total concentration of peptide was fixed at 20 µM. The fluorescent peptides were either incubated pure or “diluted” (as a mixture with their acetylated analogues).

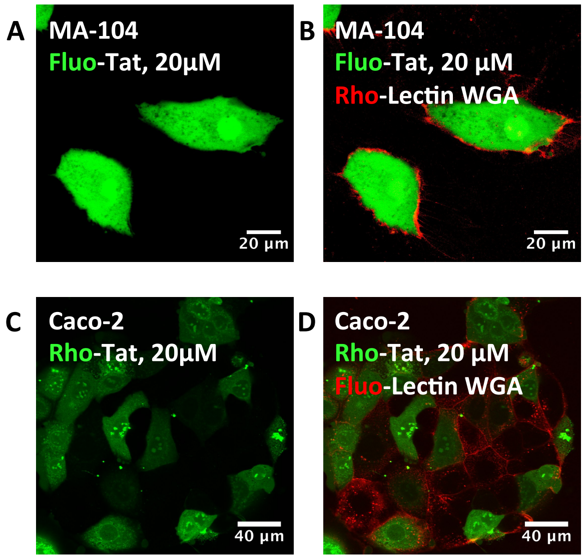

Supplementary Figure 6

The fluorescence of CPPs is quenched on cell surface.

Unfixed MA-104 or Caco-2 cells were visualized by CLSM after 10 min incubation with Fluo-Tat or Rho-Tat (20 μM) in Opti-MEM (in green) in the presence of Rho- or Fluo-Lectin WGA (0.02 mg.mL^{-1}) (in red). A and C) Fluorescence of the peptide. B and D) Overlay between the green and the red channels.

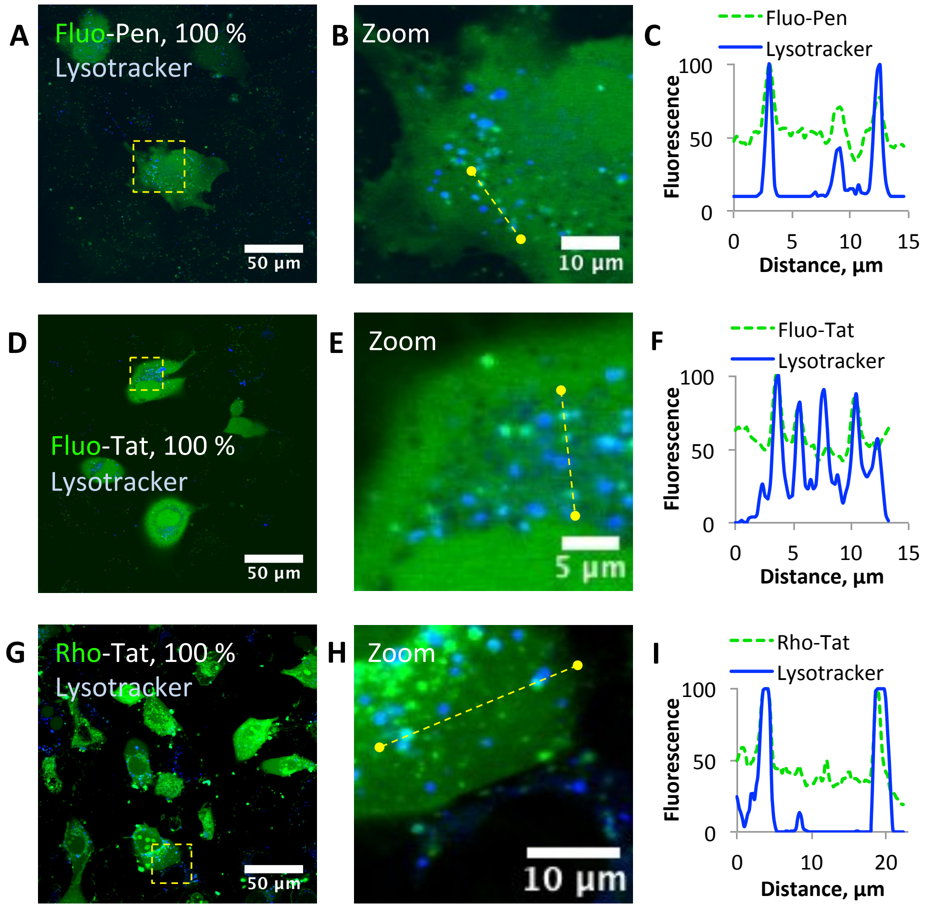

Supplementary Figure 7

Lysosomes are Pen- and Tat-rich.

Unfixed MA-104 cells were visualized by CLSM after 60 min incubation with Pen or Tat (20 µM, 100 % fluorescently labeled with fluorescein or rhodamine, in green) in Opti-MEM in the presence of lysotracker deep-red (100 nM, added 30 min before the end of the incubation, in blue). A, D and G) Fluorescence of the peptide and of the lysotracker. B, E and H) Respective enlarged views of the yellow square drawn on the picture A, D and G. C, F and I) Intensity profile of the green and blue fluorescence along the yellow cross-section respectively of the picture B, E and H.

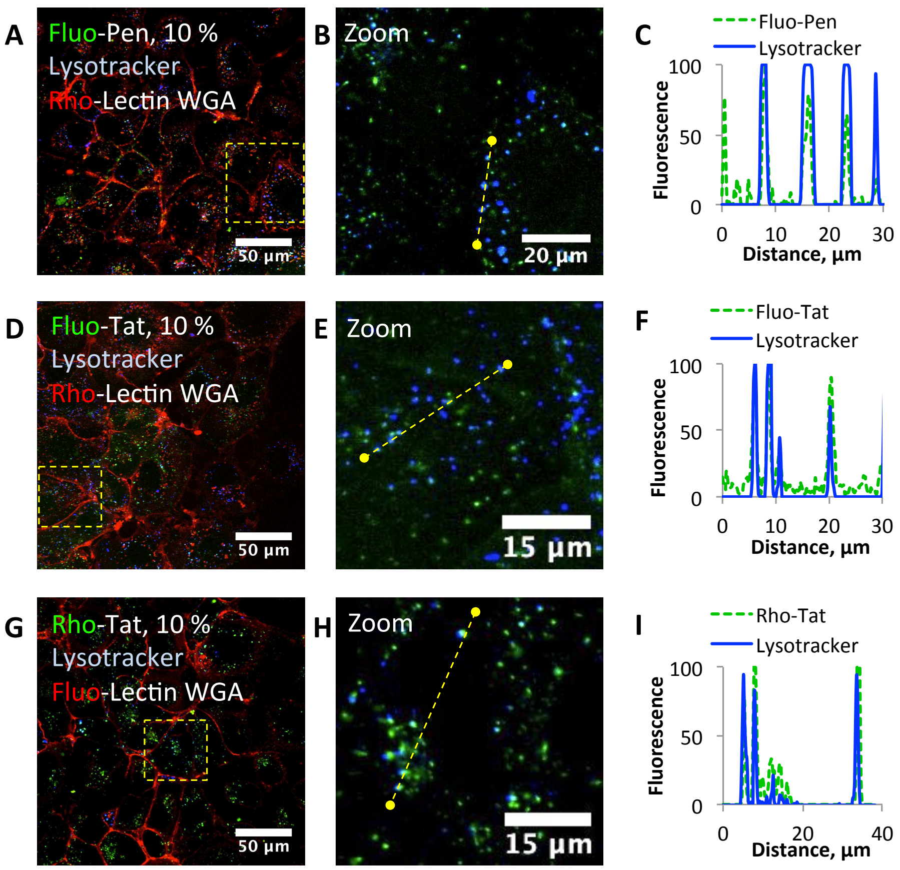

Supplementary Figure 8

The dilution experiment confirms that lysosomes are Pen- and Tat-rich.

Unfixed MA-104 cells were visualized by CLSM after 60 min incubation with Pen or Tat (20 µM, 10 % fluorescently labeled with fluorescein or rhodamine, in green) in Opti-MEM in the presence of fluorescent Lectin WGA and of lysotracker deep-red (100 nM, added 30 min before the end of the incubation, in blue). A, D and G) Fluorescence of the peptide, of the lysotracker and of the Lectin WGA. B, E and H) Respective enlarged views of the yellow square drawn on the picture A, D and G. The fluorescence of the Lectin WGA has been omitted for clarity purpose. C, F and I) Intensity profile of the green and blue fluorescence along the yellow cross-section respectively of the picture B, E and H.

Supplementary Video 1

Live fluorescence dequenching of CPPs on cell surface.

Unfixed MA-104 cells were incubated 10 min with Fluo-Arg9 (1 µM) in Opti-MEM in the presence of Rho-Lectin WGA. Cells were washed three times with Opti-MEM prior to any observation. At t = 0 min, a large excess of Ac-Arg9 (19 µM) was added and cells were live imaged in a controlled chamber (37 °C under an atmosphere of 5 % CO_2 in air). The fluorescence of the peptide is depicted in green and the one of the Lectin WGA is in red.

Supplementary Note 1

In-solution or supported syntheses

S1.1. Material and general methods

S1.1.1. Origin and nature of the reagents, solvents and reaction vessels

In-solution organic syntheses were carried out in commercially available dry solvents under anhydrous conditions (dry flasks, argon atmosphere) except if the reaction was performed in water/organic solvent mixtures. Solid-supported syntheses were performed in fritted polypropylene Torviq syringes (for manual synthesis) or in the appropriate reaction vessel (for automated peptide synthesis). Moreover, all reactions involving a fluorescent substrate were carried out in containers covered with aluminium foil.

Amino acids, MBHA resin (100-200 mesh, 0.51 or 0.43 $mmol.g^{-1}$), 1-hydroxybenzotriazole (HOBt), 2-(1H-benzotriazol-1-yl)-1,1,3,3-tetramethyluronium hexafluorophosphate (HBTU) and dicyclohexylcarbodiimide (DCC) were purchased from Iris Biotech GmbH or Novabiochem. Dry solvents and the other reagents were purchased from Sigma-Aldrich and used without further purification. Solvents for organic and peptide synthesis, MeCN for HPLC, TFA and DIPEA were purchased from Carlo Erba. Milli-Q water was produced using a Milli-Q Integral water purification system (Merck Millipore; the electrical resistivity of the water was measured and was always above 18 MΩ.cm). $CDCl_3$ was purchased from Euriso-Top.

S1.1.2. Procedure to follow progression and completion of the reactions

Progression of the in-solution reactions was monitored on analytical TLC commercially available precoated aluminium packed plates (Merck Kieselgel 60 F254). Chromogenic products were directly observed on TLC plates.

Completion of the amide bond formation on solid support was monitored with the Kaiser test. Propargylglycine does not generate the apparition of the expected blue coloration of the beads upon deprotection but a light red coloration.

S1.1.3. General procedure for automated peptide synthesis

Peptides were partially synthesized using an ABI 433A automated peptide synthesizer from Applied Biosystem in a 0.1 mmol scale in Boc strategy. A 10-fold molar excess of amino acid was used (1 mmol) with the classical HOBt/DCC activation protocol.

Peptides were manually synthesized in various scales by Boc strategy using the following protocol: amino acids (5 eq.) were activated using HBTU (4.5 eq.) and DIPEA (10 eq.) in NMP (in an appropriate volume to have an amino acid concentration of 0.25 M) over 5 min. The coupling reaction was performed in a Torviq syringe on a rotatory shaker over 30 min. The solvent and the excess of reagent were removed and the resin was washed with NMP (3 x) and CH_2Cl_2 (3 x). Some beads were transferred to a small test tube in order to perform the Kaiser test. If the used test was negative, it was followed by the deprotection of the terminal amine with TFA (1 x 10 s and 2 x 1 min). The resin was washed with CH_2Cl_2 (3 x), NMP (3 x), neutralized with 10 % DIPEA in NMP (2 x) and washed with NMP (3 x). If the Kaiser test was positive, the removed activated amino acid solution was reacted again with the peptidyl-resin over 30 min. If necessary, a double coupling with the freshly activated residue and/or a capping reaction were performed. For the capping reaction, a solution of 10 % Ac_2O in NMP was prepared containing DIPEA (10 eq.). The capping reaction proceeded over 10 min on the rotatory shaker. The next cycle is then started. A complete cycle is usually about 45 min long.

Fmoc-Pra-OH and Boc-Lys(Fmoc) were coupled manually in smaller quantities (2 eq.; the proportions of the other reagents were proportionally reduced).

Acetylation of the free *N*-terminal amine was performed by treatment with 20 % Ac_2O and DIPEA (20 eq.) in NMP for 20 min. The resin was then washed with NMP (3 x).

The derivatization of the peptides with fluorophores will be introduced below.

S1.1.4. Cleavage and work-up procedures

For Penetratin, which contains formyl protected tryptophan residues, a sequential removal was used with washes of 1, 3, 5, 7, 15, 30 min and 1 hour with a 20 % piperidine solution in DMF.

At the end of the synthesis, the peptidyl-resin was washed with NMP (3 x), CH_2Cl_2 (3 x) and MeOH (3 x) and dried under *vacuum*. The weight of the peptidyl-resin was measured and compared with the expected yield to anticipate any eventual truncation of the sequence.

Peptides were fully deprotected and cleaved from the resin by treatment with HF (2 h, 0°C) using anisole (1.5 $mL.g^{-1}$ peptidyl-resin) and methylsulfide (0.25 $mL.g^{-1}$ peptidyl-resin) as scavengers. After cleavage, the peptide was precipitated into cold diethyl ether. The precipitate was filtrated and washed with cold ether and finally dissolved in 10 % AcOH in water. The crude peptide was lyophilized.

Peptides were lyophilized using an Alpha 2/4 Freeze dryer from Bioblock Scientific.

S1.1.5. Purification materials (preparative RP-HPLC)

Peptides were purified by semi-preparative or preparative RP-HPLC using the following solvents: A = (0.1 % TFA in H_2O) and B = (0.1% TFA in MeCN) on various RP-HPLC systems. Prior to purification, crude peptides were dissolved in solvent A. Detection wavelengths were set at 220 nm (amide bond), 280 nm (tryptophan residue), 494 nm (fluorescein derivative) or 510 nm (rhodamine derivative). Semi-preparative RP-HPLC was performed using either a SymmetryPrep C8 column (7.8 x 300 mm) at a flow rate of 5.0 $mL.min^{-1}$ on a Waters system consisted of a binary pump (Waters 1525) and a dual wavelength UV/visible absorbance detector equipped with a preparative cell (Waters 2487). Preparative RP-HPLC was performed using a Nucleodur C18 column (25 x 16 mm) at a flow rate of 14.0 $mL.min^{-1}$ on a Waters system consisting of a binary pump (Waters 2535) and a dual wavelength UV/visible absorbance detector equipped with a preparative cell (Waters

2489). The software Breeze controlled all Waters systems. Peptides were analyzed by analytical RP-HPLC (see below). If necessary, RP-HPLC was repeated in order to obtain peptides in an overall purity higher than 95 %.

All peptides were finally concentrated under *vacuum* and lyophilized.

S1.1.6. Analytical material (analytical RP-HPLC, mass spectrometry and NMR analysis)

The peptides were analyzed by RP-HPLC using the following solvents: A = (0.1 % TFA in H_2O) and B = (0.1% TFA in MeCN) on a Waters System. Detection wavelengths were set at 220 nm (amide bond), 280 nm (tryptophan residue), 494 (fluorescein derivative) and/or 510 nm (rhodamine derivative). Analytical RP-HPLC was performed either using an Ace 5 C8-300 or C18-300 column (4.6 x 250 mm) at flow rate of 1.0 $mL.min^{-1}$ on a Waters systems consisting of a binary pump (Waters 1525) and a dual wavelength UV/visible absorbance detector equipped with a analytical cell (Waters 2487).

The peptides (over 95 % purity) were characterized by MALDI-TOF MS with a DE-Pro spectrometer in the positive ion reflector mode (Applied Biosystem) using α-cyano-4-hydroxycinnamic acid (HCCA) as the matrix.

NMR spectra were obtained on a Bruker 300 MHz NMR spectrometer. Chemical shifts (δ) are reported in parts per million (ppm), referenced to TMS (0 ppm). Coupling constants (J) are recorded in Hz and significant multiplicities described by singlet (s), doublet (d), triplet (t), quadruplet (q), broad (br), multiplet (m) or doublet of doublets (dd).

S1.2. In-solution synthesis of rhodamine B azide

The synthesis of Rhodamine B azide starts with the preparation of 3-azidopropan-1-ol (Supplementary Fig. 1).

Sodium azide (2.3 g, 35 mmol, 5 eq.) was added to a solution of 3-bromopropan-1-ol (1.0 g, 7 mmol) in water (15 mL). The solution was stirred overnight at 90 °C. CH_2Cl_2 (20 mL) was added and the

aqueous layer was extracted twice with CH_2Cl_2 (20 mL each time). The combined organic phases were dried over magnesium sulfate, filtrated and concentrated. 3-azidopropan-1-ol was obtained as colorless oil (580 mg, 82 % yield).

^{1}H NMR (300 MHz, $CDCl_3$): δ 3.73 (t, J = 6.5 Hz, 2H, Hc), 3.45 (t, J = 6.6 Hz, 2H, Ha), 2.47 (br, 1H, Hd), 1.85 (m, 2H, Hb).

^{13}C NMR (75 MHz, $CDCl_3$): δ 59.73 (Cc), 48.4 (Ca), 31.4 (Cb).

Rhodamine B azide (Supplementary Fig. 1) is obtained by esterification between Rhodamine B and 3-azidopropan-1-ol in the presence of EDC (1-Ethyl-3-(3-dimethylaminopropyl)carbodiimide) as activating agent. 3-azidopropan-1-ol was used without further purification.

Rhodamine B (626 mg, 1.3 mmol) and 3-azidopropan-1-ol (200 mg, 2.0 mmol, 1.5 eq.) were dissolved in 10 mL of dry CH_2Cl_2. EDC.HCl (380 mg, 2 mmol, 1.5 eq.) and DMAP (160 mg, 1.3 mmol, 1 eq.) were added to the solution. The reaction mixture was stirred overnight at RT (room temperature) and washed with 1 M HCl (2 x 10 mL), 1 M $NaHCO_3$ (2 x 10 mL) and brine (2 x 20 mL). The organic phase was dried over magnesium sulfate, filtrated and concentrated. The red oily residue was solubilized in 2 mL THF, precipitated into 50 mL of diethylether and filtrated. This operation was repeated twice to afford rhodamine B azide as a bright iridescent solid (580 mg, 75 % yield).

R_F = 0.35 (90 % CH_2Cl_2, 10 % MeOH)

^{1}H NMR (300 MHz, $CDCl_3$): δ 8.23 (d, 1H, J = 9 Hz, H6), 7.73 (m, 2H, H5+H4), 7.25 (d, J = 9 Hz, 1H, H3), 7.01 (d, J = 9 Hz, 2H, H9+H18), 6.87 (dd, J = 9 Hz and J = 3 Hz, 2H, H10+H17), 6.75 (d, J < 3 Hz, 2H, H12+H15), 4.06 (t, J = 6 Hz, 2H, Hc), 3.59 (q, J = 6 Hz, 8H, H20+H22+H24+H26), 3.17 (t, J = 6 Hz, 2H, Ha), 1.68 (tt, J = 6 Hz, 2H, Hb), 1.26 (t, J = 6 Hz, 12H, H21+H23+H24+H27).

^{13}C NMR (75 MHz, $CDCl_3$): δ 164,92 (C1), 158.63 (C7), 157.70 (C16), 155.56 (C11), 133.53 (C2), 133,28 (C4), 131,37 (C6), 131.27 (2C, C9+C18), 130.46 (2C, C3+C6), 129.2 (2C, C13+C14),

114,39 (2C, C10+C17), 113.47 (2C, C8+C19), 96.35 (2C, C12+C15) 62.54 (Ca), 48.01 (Cc), 46.21 (4C, C20+C22+C25+C26), 27.88 (Cb), 12.66 (4C, C21+C23+C24+C27).

m/z (MALDI-TOF) $[M]^{+}_{calcd}$ = 526.28 $[M]^{+}_{found}$ =526.19.

S1.3. Derivatization of peptides with the fluorescent reporters

S1.3.1. Labeling the peptides with fluorescein

5,6-carboxyfluorescein was added on the lateral side chain of a lysine residue (Supplementary Fig. 2). The peptides labeled with fluorescein were thus synthesized by Boc strategy and an extra Boc-Lys(Fmoc)-OH protected amino acid was added at the *N*-terminus sequence. Deprotection of the lateral side chain of the lysine after addition of this residue to the peptidyl-resin affords the required free amine for fluorescein labeling.

After Fmoc removal, 5,6-carboxyfluorescein (2.5 eq.) was preactivated over 10 min in presence of DIC (2.5 eq.) and HOBt (2.5 eq.) in NMP. The activated fluorescein was directly added to the peptide in a fritted syringe wrapped in aluminum foil and stirred overnight on a rotary shaker. The resin was then washed with NMP (3 x), and piperidine in NMP (20 %, v/v; 20 x 1 min, 4 x 5 min, 3 x 15 min) in order to remove polymerized 5,6-carboxyfluorescein. The resin is finally washed with CH_2Cl_2 (3 x), MeOH (3 x) and dried under *vacuum*.

S1.3.2. Click reaction protocol to obtain rhodamine labeled peptides

The click reaction with rhodamine B azide (Supplementary Fig. 2) was performed on solid support using the following protocol.

To the dry peptidyl-resin containing a propargylglycine residue, were added $Cu(OAc)_2$ (40 eq.), ascorbic acid (40 eq.) and a solution containing rhodamine B azide (20 eq.), DIPEA (50 eq) in a solution of 30% pyridine in DMF in a fritted syringe. The solution was sonicated for 5 minutes and shaken overnight at RT. The resin was then washed with DMF (3 x), DMF/Pyridine (6/5 v/v)

containing ascorbic acid (0.02 g.mL^{-1}) (3 x), CH_2Cl_2 (3 x) and MeOH (3 x). The resin was dried under *vacuum*.

S1.4. Peptide sequences and characterization of the studied peptides

The peptide sequences and their analytical characterizations are reported in Supplementary Table 2 and Supplementary Table 3, respectively.

Supplementary Note 2

The multiple origins of fluorescence self-quenching

The goal of this note is to describe concisely the possible origins of fluorescence self-quenching in our system. In particular, we would like to interpret the evolution of the fluorescence of the CPPs in the presence of an excess of DOPG (Fig. 1 and Supplementary Fig. 3). In such a case, the CPP is adsorbed and confined in the phospholipid phase and is getting progressively diluted upon addition of DOPG.

S2.1. Note on the inner-filter effect

The order of magnitude of the number of vesicle encountered by a photon emitted from a fluorescent peptide on a vesicle, noted n, is:

$$n \sim L \times \frac{a^2}{d^3}$$

L is the width of the cuvette (L ~1 cm), a is the radius of a vesicule (a ~100 nm) and d is the distance between two vesicles. Because d is defined as the distance between two vesicles, $\frac{1}{d^3}$ is the amount of vesicles per m^3. The phospholipid concentration that corresponds to the maximum of fluorescence quenching is ~10 µM and the amount of phospholipids per vesicle is ~10^5. $\frac{1}{d^3} = 10^{-7} \times N_A \sim 10^{17}$ vesicles per m^3.

$$n \sim 10^{-2} \times (10^{-7})^2 \times 10^{17} \sim 10$$

As a consequence, a photon exiting a vesicle encounters, in average, <u>at least</u> a second vesicle in solution.

S2.2. The Stern and Volmer phenomenological quenching model

The following discussion is based on one book written by Prof. Bernard Valeur.[1]

In the presence of a fluorescence quencher Q, transient interaction between the excited fluorophore and the quencher during the lifetime of the exited state may induce dynamic quenching. Dynamic quenching is usually well-described by the Stern-Volmer equation:

$$\frac{I_0}{I} = 1 + \frac{k_q}{k_f + k_{nf}}[Q] = 1 + K_{SV}[Q]$$

I_0 and I are respectively the fluorescence intensity in the absence and in the presence of the quencher. K_{SV} is the Stern-Volmer quenching constant, and [Q] the concentration of the quencher. To determine the Stern-Volmer quenching constant, I_0/I is plotted against [Q] and should yield a linear curve, whose slope is K_{SV}.

If the fluorescence quencher is a fluorophore F:

$$\frac{I_0}{I} = 1 + K_{SV}[F]$$

Fluorescence energy transfer between two identical fluorophores cannot be the physical origin of dynamic self-quenching since it is not accompanied by any energy loss. In consequence, fluorescence transfers with “energy losses" have to be envisioned.

Alternatively, when the concentration of quencher is high enough, so that the quencher is always in interaction with the fluorophore, static quenching may occur. Static quenching might be due to the formation of a ground-state complex between the fluorophore and the quencher and in our case the quencher can be a fluorophore F. The equilibrium constant of the ground state complex formation, K_S, is:

$$K_S = \frac{[F-F]}{[F]^2}$$

The dependence of fluorescence intensity on the concentration of the quencher, here the fluorophore F, can be derived and gives again a linear relationship between I_0/I and [F]:

$$\frac{I_0}{I} = \frac{[F]_0}{[F]} = 1 + 2K_S[F]$$

Not surprisingly, static quenching decreases the number of fluorescent dyes inducing a global decrease of the quantum yield.

In conclusion, if classical dynamic or static quenching is responsible for the observed fluorescence decrease, linear Stern-Volmer plots will be observed.

S2.3. Phenomenology of fluorescence quenching: analysis of the titration curves

The titration protocol slightly differs from the usual quenching experiments. The concentration of the fluorophore, noted $[F]_0$, is maintained constant, but the amount of the lipid phase, noted [DOPG], changes. As a consequence, we cannot directly plot the ratio between the initial fluorescence and the fluorescence in the presence of the quencher (I_0/I) *versus* the concentration of the quencher [Q]. Equivalently, we could plot this ratio against the inverse of the concentration in DOPG. Indeed, the surface density of CPP is inversely proportional to the amount of introduced DOPG. But here, the fluorescence I_0 in the absence of quencher is also not known. This fluorescence would be the one of the CPP infinitely diluted in the lipid phase. We only know the fluorescence in the presence of a small amount of quencher. As a consequence, we will only plot the inverse of fluorescence intensity against the inverse of the concentration in DOPG.

This curve is not strictly equivalent to a Stern-Volmer plot. In a Stern-Volmer plot, the horizontal axis corresponds to the concentration of the free quencher, [Q] (fluorophore in our case, [F]). Here, the horizontal axis takes into account the total concentration of quencher (fluorophore in our case, $[F]_0$). In the case of static quenching, the true Stern-Volmer plot is a straight line, while our representation will always give a downward curvature. Our representation should always tend to a downward curvature compared to the Stern-Volmer plot, since the concentration of free fluorophore is a concave function of the total concentration of fluorophore.

At low 1/[DOPG] values, *ie.* at high dilution of the CPPs in the phospholipid phase, our plot corresponds to a linear behavior (Supplementary Fig. 3). However, when the CPP is densely packed on the surface, an upward curvature is always observed (Supplementary Fig. 3). Such a curvature

signifies that at high CPP:DOPG ratio, a very important self-quenching occurs. Because the simple dynamic or static models that we proposed do not satisfactorily describe the deviation, they have to be adapted.

The upward curvature of our plot, concave towards the y-axis may originate from three different phenomena: (i) dynamic and static quenching can occur simultaneously or (ii) the space between the fluorophore and the quencher (*i.e.* another fluorophore) is small enough for the quencher to be next to the fluorophore at the moment of the excitation –as a result, the fluorophore is immediately quenched without the requirement of the formation of a physically defined ground complex– or (iii) the static quenching yields "black dimers", which are themselves efficient quenchers. We will successively investigate these possibilities.

S2.4. How to explain the upward curvature of fluorescence self-quenching?

S2.4.1. Combined dynamic and static fluorescence quenching

We interpreted static quenching as the formation of a complex between the fluorophore and the quencher. If static and dynamic quenching occurs simultaneously, this means that dynamic quenching can decrease the fluorescence of the remaining non-complexed fluorophore. Symmetrically, this means that the formation of a non-fluorescent complex can reduce the fraction of free fluorophore that were dynamically quenched:

$$\frac{I_0}{I} = \left(\frac{I_0}{I}\right)_{dynamic} \times \frac{[F]}{[F]_0} = (1 + K_{SV}[F])(1 + 2K_S[F])$$

And if we develop this equation:

$$\frac{I_0}{I} = 1 + (K_{SV} + 2K_S)[F] + 2K_{SV}K_S[F]^2$$

This modified Stern-Volmer equation, which takes simultaneously into account static and dynamic quenchings, is second order in [F]. As a consequence, the corresponding Stern-Volmer plot has an upward curvature and could explain our observation.

However, this model does not explain the extinction of fluorescence in a CPP-rich environment. Indeed, static quenching only decreases the concentration of visible fluorophore and dynamic quenching only yields to a plateau of fluorescence, not to fluorescence extinction. So, the combination of both effects may explain these experimental curves, but not the extinction of membrane fluorescence that we observe with living cells.

S2.4.2. The quenching sphere of action model

Alternatively, static quenching can also be envisioned without the formation of a stable and defined complex. This is for example the case when the concentration is high enough and the quencher is adjacent to the fluorophore at the moment of excitation. Such pairs lead to immediate fluorescence quenching. This could be the case in our system, when the surface of the vesicles is crowded.

Perrin proposed a model of such a situation, in which fluorescence quenching is total if a quencher is inside a sphere centered on the fluorophore. This is the so-called model of "quenching sphere of action". If the quencher is outside the sphere it has no effect on the fluorophore, whereas inside it quenches the fluorescence. Assuming that the number of quencher inside a small volume follows a Poisson distribution, the ratio between the fluorescence intensity in the absence and in the presence of the quencher is:

$$\frac{I_0}{I} = \exp\left(2V_q N_A [F]\right)$$

At low concentrations, this model follows a simple Stern-Volmer relation:

$$\frac{I_0}{I} \cong 1 + 2V_q N_A [F]$$

But at high concentrations, a positive deviation from the Stern-Volmer plot is also observed.

However, this model relies on the assumption that the transfer of fluorescence occurs with some losses.

S2.4.3. Formation of "dark dimers"

This last model is based on the static fluorescence quenching and assumes that fluorophore dimers (or aggregates) can absorb light at the emission wavelength of the fluorophores. These dimers thus become "black dimers".[2] In this model, a "transfer with losses" between two fluorophores is not required: the dimers are the fluorescence quenchers.

If dimers are fluorescence quenchers, at low CPP concentration we only observe a linear plot due to formation of dimers; this is the static quenching. At a higher CPP concentration, the concentration of the dimers is high enough to induce an extra dynamic quenching of fluorescence by the dimers. At the thermal equilibrium, the concentration of dimers is roughly the square of the total concentration of CPP. As a consequence, the extra dynamic quenching induced by the dimers is proportional to the square of the total concentration. In summary, at low concentration of CPP, the plot is linear and has a positive curvature at medium concentration.

This model further explains the quantitative fluorescence extinction of the fluorescent CPP near the plasma membrane at high concentration. Indeed, at medium to high concentration, the quenching efficiency is proportional to the square of the CPP concentration: the total fluorescence decreases as the inverse of the concentration of CPP

In conclusion, the fluorescence self-quenching of CPPs on membranes can be interpreted as follows:

(i) Because of the concentration of CPPs on membrane surface, "dark dimers" of fluorophores can form. Upon light absorption, they do not emit any photon. These dimers correspond to the classical case of static quenching.

(ii) Alternatively, transient interactions between fluorophores can quench the fluorescence. This corresponds to the classical dynamic quenching.

(iii) At high CPP concentrations, the positive deviation from the Stern-Volmer plot can be explained both by the quenching sphere of action model or by a dynamic quenching by dimers themselves.

Supplementary Note 3

Determination of the cell-viability in the presence of peptides

Viability assays were performed in transparent 96-well plates (Thermo Scientific) using the CCK-8 assay (Dojindo Molecular Technologies). Viability was determined by measuring the absorbance of each well at 450 nm using a microplate reader (Molecular Devices).

MA-104 and Caco-2 cells were seeded in 96-well plates (5000 cells/well) in DMEM. The day after, cells were washed twice with Opti-MEM and incubated for 120 min with fluorescent CPPs (20 μM, either as pure fluorescent CPPs or as a mixture between acetylated CPP and fluorescent CPP, 5 %,) in Opti-MEM (100 μL) at 37 °C. Controls were performed with incubation in pure Opti-MEM (positive control) or 0.1 % SDS in Opti-MEM (negative control). The supernatant was removed and the cells were washed once with Opti-MEM. CCK-8 solution was diluted 10 times in Opti-MEM and 100 μL of this solution was distributed in each well. After 2 h incubation at 37 °C, cell viability was determined by measuring the absorbance of each well at 450 nm (Supplementary Fig. 5).

Supplementary Table 1: Sequences of the three studied CPPs and their respective affinities for glycosaminoglycans or anionic phospholipids.

Peptide	Sequence	Affinity for HS, K_d, μM[a]	Affinity for PLs, K_d, μM
Penetratin (Pen)[3]	RQIKIWFQNRRMKWKK	nd[b]	63 (DOPC/DOPG 90:10)[4]
Tat peptide (Tat)[5]	YGRKKRRQRRR	1.67[6]	83 (POPC/POPG 75:25)[7]
Arg9[8]	RRRRRRRRR	0.32[9]	12 (POPC/POPG 75:25)[9]

[a] HS: heparan sulfate. [b] nd: not determined.

Supplementary Table 2: Sequences of the peptides used in the study.

	Peptides	N°	Sequences
Ac-Peptides	Ac-Pen	**1**	Ac-RQIKIWFQNRRMKWKK-NH_2
	Ac-Tat	**2**	Ac-YGRKKRRQRRR-NH_2
	Ac-Arg9	**3**	Ac-RRRRRRRRR-NH_2
Fluo-Peptides	Fluo-Pen	**4**	Ac-K(Fluo)RQIKIWFQNRRMKWKK-NH_2
	Fluo-Tat	**5**	Ac-K(Fluo)YGRKKRRQRRR-NH_2
	Fluo-Arg9	**6**	Ac-K(Fluo)RRRRRRRRR-NH_2
Rho-Peptide	Rho-Tat	**7**	Ac-A(Rho)YGRKKRRQRRR-NH_2

Supplementary Table 3: Analytical retention times, calculated and measured m/z of the different peptides synthesized. *Ac-Pen, Ac-Tat, Ac-Arg9 and Fluo-Arg9 were characterized with an Ace 5 C8-300 column (4.6 x 250 mm). Fluo-Tat, Fluo-Pen and and Rho-Tat were analyzed on a Ace 5 C18 column.

N°	HPLC gradient (Analytical)	Retention time (min)*	Calculated m/z	Measured m/z
**1**	15 to 45 % B in 15 min	11.0	$[M+H]^+$ 2319.31	2319.60
**2**	5 to 25 % B in 20 min	11.8	$[M+H]^+$ 1600.98	1600.88
**3**	0 to 30% B in 15 min	9.2	$[M+H]^+$ 1464.95	1464.94
**4**	15 to 45 % B in 20 min	13.6	$[M+H]^+$ 2805.45	2805.29
**5**	5 to 60 % B in 20 min	12.5	$[M+H]^+$ 2087.12	2087.96
**6**	5 to 60 % B in 20 min	12.0	$[M+H]^+$ 1951.09	1951.75
**7**	20 to 80 % B in 20 min	13.5	$[M+H]^+$ 2222.30	2222.08

Supplementary references

1. Valeur, B. in *Molecular Fluorescence* 72–124 (Wiley-VCH Verlag GmbH, 2001).
2. Chen, R. F. & Knutson, J. R. Mechanism of fluorescence concentration quenching of carboxyfluorescein in liposomes: energy transfer to nonfluorescent dimers. *Anal. Biochem.* **172,** 61–77 (1988).
3. Derossi, D., Joliot, A. H., Chassaing, G. & Prochiantz, A. The third helix of the Antennapedia homeodomain translocates through biological membranes. *J. Biol. Chem.* **269,** 10444–10450 (1994).
4. Persson, D., Thorén, P. E. G., Herner, M., Lincoln, P. & Nordén, B. Application of a novel analysis to measure the binding of the membrane-translocating peptide Penetratin to negatively charged liposomes. *Biochemistry* **42,** 421–429 (2003).
5. Vivès, E., Brodin, P. & Lebleu, B. A truncated HIV-1 Tat protein basic domain rapidly translocates through the plasma membrane and accumulates in the cell nucleus. *J. Biol. Chem.* **272,** 16010–16017 (1997).
6. Hakansson, S. & Caffrey, M. Structural and dynamic properties of the HIV-1 tat transduction domain in the free and heparin-bound states. *Biochemistry* **42,** 8999–9006 (2003).
7. Ziegler, A., Li Blatter, X., Seelig, A. & Seelig, J. Protein transduction domains of HIV-1 and SIV TAT interact with charged lipid vesicles. Binding mechanism and thermodynamic analysis. *Biochemistry* **42,** 9185–9194 (2003).
8. Wender, P. A. *et al.* The design, synthesis, and evaluation of molecules that enable or enhance cellular uptake: Peptoid molecular transporters. *Proc. Natl. Acad. Sci.* **97,** 13003–13008 (2000).
9. Gonçalves, E., Kitas, E. & Seelig, J. Binding of oligoarginine to membrane lipids and heparan sulfate: structural and thermodynamic characterization of a cell-penetrating peptide. *Biochemistry* **44,** 2692–2702 (2005).
